# Development and reliability of an intra-operative first metatarsophalangeal joint cartilage evaluation tool for use in hallux valgus surgery

**DOI:** 10.1186/1757-1146-4-S1-O45

**Published:** 2011-05-20

**Authors:** Simon E Smith, Karl B Landorf, Mark F Gilheany, Hylton B Menz

**Affiliations:** 1 Department of Podiatry, Faculty of Health Sciences, La Trobe University, Bundoora, Victoria, Australia; 2 Musculoskeletal Research Centre, Faculty of Health Sciences, La Trobe University, Bundoora, Victoria, Australia; 3 East Melbourne Podiatry, East Melbourne, VIC, 3002, Australia

## Background

There is increasing interest in surgical outcomes of hallux valgus (HV) reconstruction. However, there has been little focus on the influence of cartilage degeneration that is identified within the first metatarsophalangeal joint (1st MTPJ) at surgery. A reliable evaluation tool to accurately record cartilage erosion within the 1st MTPJ is desirable, and should be part of evidence-based reporting on surgical reconstruction for HV. The objective of this study was to examine the reliability of an intra-operative evaluation tool for assessing cartilage degeneration of the 1st MTPJ in hallux valgus.

## Methods

During hallux valgus reconstruction surgery, two examiners documented the location, depth and surface area of cartilage lesions affecting the 1st MTPJ in 20 females aged 17 to 69 years (mean 50.9, SD. 13.5). Depth of cartilage lesions was assessed using the 5-level International Cartilage Repair Society (ICRS) scale and a 3-level scale (normal, partial thickness, full thickness). Inter-examiner reliability of lesion location and depth was assessed using absolute percentage agreement and kappa (*κ*) statistics, and inter-examiner reliability of lesion surface area was assessed using intra-class correlation coefficients (ICCs) and 95% limits of agreement (LOAs).

## Results

For lesion location, percentage agreement ranged from 90 to 100% and *κ* values ranged from 0.78 to 1.00, reflecting substantial to excellent levels of agreement. For lesion depth using the ICRS and 3-level scale, percentage agreement ranged from 33 to 100% and weighted *κ* values ranged from 0 to 1.00, reflecting poor to excellent levels of agreement. For lesion surface area, the ICC was 0.98 (95% CI, 0.97 to 0.99) and 95% LOA was 0.74 to 1.41, indicating excellent reliability.

## Conclusions

The results of this study demonstrate a generally high degree of reliability between examiners for the intra-operative use of the 1st MTPJ cartilage evaluation tool. The tool may have some value in predicting surgical outcomes associated with hallux valgus.

